# Bespoke design of whole‐cell microbial machines

**DOI:** 10.1111/1751-7915.12460

**Published:** 2016-11-17

**Authors:** Claudia Vickers

**Affiliations:** ^1^ Australian Institute for Bioengineering and NanotechnologyThe University of QueenslandSt LuciaQLD4072Australia

Six years ago, I wrote a perspective article with Lars Blank (now at Aachen) and Jens Kromer (The University of Queensland) describing a grand challenge of developing chassis cells: tailorable cells that can be used to rapidly engineer production of industrially useful biochemicals (Vickers *et al*., 2010). We listed four requirements to develop chassis cells: a minimal cell, with associated reduction of complexity; the ability to tightly and predictably control overall cellular behaviour; the ability to precisely direct cellular carbon flux towards desired products; and a toolbox of technologies that enable high‐level microbial engineering. Here, I will examine progress in each of these areas. I will also speculate on where we might go with this technology in the future.

The minimal genome developed recently by the J. Craig Venter Institute (JCVI; (Hutchison *et al*., 2016) is the smallest known genome capable of sustaining self‐replication of a free‐living organism – albeit one that grows relatively slowly and that requires fairly complex nutritional support. Significant work is required to develop an industrially useful chassis cell using this technology, including improved growth rate and the ability to grow well under stresses typical of an industrial bioprocess (Vickers, 2016). However, it does demonstrate proof of concept for extreme genome minimization – one of the two approaches to construct a chassis cell. The other is greenfield genome design, a considerably more challenging approach requiring both the capacity to synthesize complete genomes and a full understanding of minimal metabolic requirements. While writing DNA has notably lagged behind reading DNA, we are now at the stage where, with a reasonable amount of resources and infrastructure, one can write entire microbial genomes from templates. However, of the 473 genes encoded on the minimal genome, the function of 149 is currently unknown (Hutchison *et al*., 2016) – indicating that we still have some way to go to achieve the sufficiently detailed understanding of cellular requirements that would enable true greenfield design. Notwithstanding this, it is fair to say that we are starting to move towards the point where we can seriously consider the ground‐up construction of chassis cells. This will be accelerated by the ability to interrogate in detail what it takes to make a functional genome through the kind of genome minimization experiments pioneered at JVCI.

The second requirement for chassis cell design is the ability to tightly and predictably control overall cellular behaviour. This goes hand in glove with the detailed understanding of metabolic behaviour required to build a cell from the ground‐up. Understanding how regulatory circuits control native cellular behaviour is of course only the first step; an ability to exploit those regulatory circuits will be required to fully realize the potential of a chassis cell system. Synthetic biology approaches for the construction of discrete genetic circuitry are paving the way towards more complex and broad‐reaching regulatory control, and orthologous methods – which are developing rapidly – will undoubtedly be required to obtain full control over cellular behaviour.

Our ability to precisely direct cellular carbon flux towards desired products has come a long way over the last handful of years. In particular, metabolic modelling approaches – including an improved ability to incorporate kinetic information into models (Saa and Nielsen, 2016) – have progressed significantly. In the future, such models might start to provide a foundation for greenfield genome design. Experimentally, the ability to control carbon flux at specific metabolic nodes using a variety of different approaches and, perhaps more importantly, the ability to balance metabolic flux across metabolic pathways and within the entire metabolic network (aided by modelling) are improving rapidly. Key to this is understanding metabolic fluxes on a global cellular scale. While we have come a long way in our capacity to measure fluxes, challenges still remain in this area (see ‘Let′s talk about flux’, this issue by Lars Blank).

Finally, our toolbox of enabling technologies has advanced significantly in recent years. *Escherichia coli* and yeast toolboxes are very well developed, and other organism toolboxes are catching up. As we discussed previously (Vickers *et al*., 2010), the development of an easily accessible central database of information linked to strain and part libraries will be very useful. The expansion and consolidation of collective databases (including GenBank, MetaCyc, BRENDA and SGD) with links to part facilities (such as AddGene, the Coli Genetic Stock Centre, and other plasmid/strain repositories) would help support engineering efforts. In terms of actual chassis cells, designs for both *E. coli* (Umenhoffer *et al*., 2010) and yeast (Jouhten *et al*., 2016) are underway, and will most likely move forward quickly in combination with the JCVI genome minimization approach. Perhaps the most advanced industrial production chassis currently in use is the C15 isoprenoid chassis developed by the industrial bioscience company Amyris. While it is not a genome‐reduced chassis, it is very efficient; in combination with Amyris’ robotic strain development platform, it can be used to rapidly achieve high gram/L production titres.

I envisage a future where metabolic engineers have a one‐stop shop for microbial cell factory chasses that have specific base pathway augmentations (e.g., isoprenoid boosted, shikimate boosted, phosphoketolase boosted and non‐ribosomal peptide boosted) and can be tailored rapidly to provide deployable production strains. That is the first generation; the next generation will be a broader selection of pathway‐specific strains; for example, isoprenoid production strains tailored for C5, C10, C15, C20, etc.; and the third generation will be more specific sub‐categories, e.g. carotenoids, tetraterpene derivatives, sterol pathway intermediates. The shop will include a variety of tailored decoration enzymes to modify/remove/add moieties – P450s, methyltransferases, oligotransferases, glycosyltransferases, etc.; these enzymes will be rapidly engineerable, have high specific activity, and lack regulatory controls. Modular toolkits to develop bolt‐on parts are available, including a recently described yeast kit (Lee *et al*., 2015). In the future, we will also have a wide variety of validated bolt‐on modules, including regulatory componentry (based on transcription factor sets and global response cascades), product export componentry and titre‐boosting componentry – all of which will require minimal tailoring for the product of interest.

The future of the microbial cell factory industry will be underpinned by rapid advances in computer‐aided design, genome construction and high‐throughput multiplexed robotic strain development/testing. Powerful new tools such as CRISPR will also play an important role. CRISPR is a game‐changing technology from two respects. First, the remarkable power of the technology for genome editing is already delivering applied results in diverse fields. In the context of development of microbial cell factories this provides the potential for rapid retro fitting of the already‐developed chassis for closely related applications. Second, CRISPR has broken new ground in regulatory legislation. In the United States, the decision has been taken that a CRISPR‐modified organism is not a GMO, since no foreign DNA has been introduced into the cell. This likely means that the deployment of CRISPR‐modified engineered organisms can happen far more rapidly, once initial approval has been provided for the original chassis.

Our current limitation remains our basic understanding of cellular biology and, in particular, our ability to rapidly and predictably engineer that biology. However, that understanding is expanding very quickly, and as it does, we accelerate towards our ultimate goal to achieve bespoke design and implementation of whole‐cell microbial machines. Eventually, we may have a design‐build‐deploy capacity for microbial machines (Vickers, 2016) that will allow us to rapidly deliver economically competitive bioprocesses. This will help us build a more sustainable future through the use of renewable feedstocks – currently agricultural products, but ultimately waste materials (e.g. gas fermentation) and direct photosynthesis (e.g. using cyanobacteria) – to produce the vast array of chemicals required to support the modern lifestyle of humanity.
